# Supplementary material for: A metasomatized lithospheric mantle control on the metallogenic signature of post-subduction magmatism
Source: Nat Commun. 2019 Aug 5;10:3511. doi: 10.1038/s41467-019-11065-4 (PMC6683204; doi:10.1038/s41467-019-11065-4)
Supplement: Supplementary file 2 — Description of Additional Supplementary Files [file 41467_2019_11065_MOESM2_ESM.pdf]

Description of Additional Supplementary Files

File name: Supplementary Data 1

Description: Bulk rock major element and chalcophile geochemistry of samples used in this study. Note that major element data for Sron Garbh data are not available. Chalcophile data were obtained through a trace element assay package by Scotgold Resources that did not include major elements.
