# Supplementary material for: A metasomatized lithospheric mantle control on the metallogenic signature of post-subduction magmatism
Source: Nat Commun. 2019 Aug 5;10:3511. doi: 10.1038/s41467-019-11065-4 (PMC6683204; doi:10.1038/s41467-019-11065-4)
Supplement: Supplementary file 3 — Supplementary Data 1 [file 41467_2019_11065_MOESM3_ESM.pdf]

A metasomatized lithospheric mantle control on the metallogenic signature of post-subduction magmatism

Holwell et al.

Supplementary Data

Location	Sample ID	Zone	Rock type	SiO2 wt%	TiO2 wt%	Al2O3 wt%	Fe2O3 wt%	MnO wt%	MgO wt%	CaO wt%	Na2O wt%	K2O wt%	P2O5 wt%	SO3 wt%	V2O5 wt%	Cr2O3 wt%	SrO wt%	ZrO2 wt%	BaO wt%	NiO wt%	CuO wt%	ZnO wt%	PbO wt%	LOI wt%	Total wt%	ppm Ni	ppm Co	ppb Ir	ppb Ru	ppb Rh	ppb Pt	ppb Pd	ppb Au	ppm Cu	ppm Te
BAL1	Mantle	Balmuccia, Italy	Spinel harzburgite	38.42	0.01	0.61	10.08	0.136	46.83	0.338	<0.02	<0.01	0.004											2.64	99.05	2463.4	124.11	5.49	10.7	1.9	6.22	2.23	0.3	3.5	0.007
BAL2	Mantle	Balmuccia, Italy	Spinel harzburgite	43.94	0.06	2.77	8.93	0.134	40.48	2.716	0.06	<0.01	<0.002											0.46	99.55	1902.6	99.45	3.94	7.2	1.3	6.91	6.2	1.11	23.7	0.007
BAL3	Mantle	Balmuccia, Italy	Spinel harzburgite	44.11	0.05	2.55	9.04	0.131	41.61	2.304	0.04	<0.01	<0.002											-0.03	99.8	2003.7	101.95	4.26	7.93	1.4	7.47	6.86	1.24	23.5	0.007
BAL4	Mantle	Balmuccia, Italy	Spinel harzburgite	43.49	0.05	1.64	8.66	0.122	43.50	1.238	0.04	0.01	0.004											0.65	99.41	2165.4	109.75	4.46	7.49	1.12	4.55	2.84	0.78	16.5	0.004
BAL6	Mantle	Balmuccia, Italy	Lherzolite vein	52.21	0.16	4.37	5.9	0.134	24.36	11.038	0.32	0.02	0.002											0.31	98.81	797.9	49.46	2.04	1.41	3.09	51.4	134	13.7	117.7	0.050
BAL8	Mantle	Balmuccia, Italy	Pyroxenite vein	50.79	0.11	3.14	5.41	0.119	24.20	13.832	0.34	<0.01	<0.002											0.29	98.22	952.2	51.63	2.45	1.54	1.63	97.3	152	41.2	159.3	0.050
XM1/142-A	Mantle	Bultfontein, RSA	Phlogopite-spinel lherzolite	46.71	0.12	0.93	6.04	0.099	39.58	0.689	0.07	0.81	0.051											4.35	99.45	1309	63.0	4.08	7.48	1.20	3.87	0.44	0.56	3.5	0.017
XM1/142-B	Mantle	Bultfontein, RSA	Phlogopite-spinel lherzolite	46.3	0.94	2.69	6.52	0.088	34.43	2.278	0.28	2.45	0.055											3.77	99.8	1123	61.9	3.58	11.80	1.72	5.81	1.21	2.01	27.5	0.019
XM1/341	Mantle	Bultfontein, RSA	Phlogopite-spinel lherzolite	43.47	0.33	0.93	8.67	0.121	39.53	1.015	0.07	0.9	0.045											4.81	99.89	1635	91.2	5.95	3.22	0.62	2.96	0.93	1.62	22.2	0.113
XM1/345	Mantle	Bultfontein, RSA	Phlogopite-spinel lherzolite	41.55	0.31	1.48	9.68	0.116	40.99	0.38	<0.02	1.47	0.049											3.64	99.67	1885	113.8	3.07	6.57	0.47	0.59	0.65	0.77	5.5	0.017
XM1/355	Mantle	Bultfontein, RSA	Garnet harzburgite	42.08	0.02	1.65	5.96	0.087	44.59	0.367	<0.02	0.04	0.028											5.14	99.96	1640	81.9	2.31	4.19	0.72	0.65	0.25	0.52	4.4	0.016
XM1/422	Mantle	Bultfontein, RSA	Spinel harzburgite	44.44	0.03	0.93	6.49	0.095	44.39	0.304	<0.02	0.02	0.014											3.52	100.23	1721	80.2	3.87	4.73	0.60	0.66	0.17	0.46	3.3	0.017
V-LZD2	Lower crust	Valmaggia, Italy	Amphibole-phlogopite lherzolite	35.98	0.84	8.02	23.23	0.16	18.07	5.29	0.89	0.46	0.376	0.151	0.028	0.163	0.011	0.028	0.074	0.833	0.149	0.013	0.003	4.09	98.86	4570.3	255.0				15.8	279.7	11.3	830	0.732
V-LZAB	Lower crust	Valmaggia, Italy	Amphibole-phlogopite lherzolite	36.19	0.40	7.82	24.75	0.25	23.48	3.07	0.50	0.23	0.090	0.475	0.020	0.175	0.007	0.009	0.054	0.550	0.244	0.022	0.003	2.28	100.63	2815.9	231.0					32.1		1299	0.286
V-LZD1A	Lower crust	Valmaggia, Italy	Amphibole-phlogopite lherzolite	38.19	0.95	9.00	19.92	0.16	18.17	6.07	1.09	0.41	0.214	0.072	0.031	0.172	0.010	0.028	0.060	0.665	0.152	0.013	0.003	3.59	98.97	4080.0	205.1				39.4	322.0	9.5	997	0.505
V-LZD1B	Lower crust	Valmaggia, Italy	Amphibole-phlogopite lherzolite	39.13	0.87	10.45	18.63	0.17	18.27	6.41	1.17	0.43	0.221	0.140	0.028	0.172	0.011	0.027	0.070	0.520	0.217	0.018	<0.002	2.88	99.84	3109.9	176.3				12.1	263.8	31.3	1395	0.297
V-LZ-D1C	Lower crust	Valmaggia, Italy	Amphibole-phlogopite lherzolite	39.23	0.94	9.24	18.64	0.17	18.85	6.08	1.08	0.52	0.379	0.236	0.031	0.178	0.010	0.038	0.111	0.516	0.116	0.016	<0.002	2.87	99.24	2981.5	169.6				11.3	230.9	7.5	718	0.229
V-L2A	Lower crust	Valmaggia, Italy	Amphibole-phlogopite lherzolite	37.52	0.36	6.87	21.48	0.25	24.62	3.69	0.63	0.27	0.111	0.638	0.017	0.144	0.014	0.014	0.072	0.281	0.075	0.026	0.002	1.10	98.19	2021.7	168.6					83.1		544	0.139
V-L2B	Lower crust	Valmaggia, Italy	Amphibole-phlogopite lherzolite	49.68	0.25	5.41	15.76	0.21	25.81	1.41	<0.015	0.06	0.002	0.189	0.044	0.185	<0.004	<0.002	<0.036	0.059	0.258	0.020	<0.002	0.50	99.84	881.8	93.8					94.0	10.5	740	0.116
V-L2C	Lower crust	Valmaggia, Italy	Amphibole-phlogopite lherzolite	41.79	0.43	16.01	11.65	0.16	14.69	10.12	1.23	0.20	0.069	0.650	0.018	0.047	0.044	0.004	<0.036	0.128	0.064	0.011	<0.002	1.57	98.88	727.0	64.8				6.09	69.0		400	0.099
V-L3B	Lower crust	Valmaggia, Italy	Amphibole-phlogopite lherzolite	44.64	0.38	21.66	8.64	0.10	8.22	10.63	2.34	0.22	0.113	0.052	0.013	0.007	0.058	0.005	0.043	0.087	0.076	0.007	<0.002	1.05	98.34	516.6	39.4					63.9		455	0.174
VMG2	Lower crust	Valmaggia, Italy	Amphibole-phlogopite lherzolite	40.07	0.51	7.31	16.1	0.209	23.59	8.176	0.89	0.08	0.046											1.56	98.55	1183.9	154.3	0.12	0.13	0.12	0.27	3.08	2.55	415.6	0.070
VMG5	Lower crust	Valmaggia, Italy	Amphibole-phlogopite lherzolite	48.34	0.54	19.59	6.84	0.101	8.18	12.715	2.52	0.2	0.059											1.16	100.25	209.70	34.65	0.02	<0.08	0.02	<0.17	0.17	0.80	99.40	0.016
VMG6	Lower crust	Valmaggia, Italy	Amphibole-phlogopite lherzolite	47.75	0.68	20.23	6.91	0.105	6.72	12.907	2.69	0.25	0.055											1.68	99.98	421.90	44.93	0.08	<0.08	0.10	1.18	1.60	2.28	321.90	0.060
VMG7	Lower crust	Valmaggia, Italy	Amphibole-phlogopite lherzolite	39.46	1.00	5.06	18.48	0.245	28.96	3.058	0.94	0.32	0.148											1.32	98.99	572.80	116.32	0.04	<0.08	0.04	0.23	0.38	0.39	37.20	0.013
I7	Lower crust	Valmaggia, Italy	Amphibole-phlogopite lherzolite	37.01	0.70	7.97	21.9	0.128	19.39	5.25	0.87	0.35	0.15											4.7	98.42	>4100	>187	18.11	22.20	6.98	26.50	69.30	10.10	863.90	0.950
I2	Lower crust	Valmaggia, Italy	Amphibole-phlogopite lherzolite	35.75	0.36	1.85	17.11	0.267	29.67	2.823	<0.02	0.02	0.029											10.92	98.76	1975.9	155.26	0.38	4.12	0.25	1.82	2.51	3.64	129.00	0.018
FDD1	Lower crust	Valmaggia, Italy	Amphibole-phlogopite lherzolite	42.67	1.18	9.03	16.69	0.208	21.20	4.764	1.48	0.76	0.326											1.14	99.44	2131.3	116.33	0.88	0.86	0.43	17.50	5.03	10.40	654.10	0.290
FDD1A	Lower crust	Valmaggia, Italy	Amphibole-phlogopite lherzolite	42.64	1.09	9.79	15.3	0.203	20.32	5.004	1.58	0.73	0.294											1.57	98.53	1526.0	98.56	0.47	0.51	0.28	5.11	3.94	9.01	490.40	0.170
SGAQ14-13	Mid crust	Sron Garb, Scotland	Appinite																							1602.0	276.1				410.2	563.3	185.0	6115.1	0.288
SGAQ15-06	Mid crust	Sron Garb, Scotland	Appinite																							1022.8	100.7				242.6	301.6	32.1	3777.5	0.268
SGAQ15-07	Mid crust	Sron Garb, Scotland	Appinite																							2715.8	225.7				514.9	627.0	146.8	8840.9	0.638
SGAQ16-12	Mid crust	Sron Garb, Scotland	Appinite																							1800.0	132.1				380.9	755.8	158.7	5783.8	0.768
SGAQ16-13	Mid crust	Sron Garb, Scotland	Appinite																							1720.5	90.9				774.2	794.2	275.3	9151.8	1.208
G318-4	Upper crust	Gangdese, China	Amphibolite	56.7	0.73	16.5	8.35	0.19	3.9	7.41	2.7	0.95	0.12											0.28	97.88	10	16				5.4	6	6		0.030
BR-1	Upper crust	Gangdese, China	Strongly altered porphyry	68.3	0.4	15.35	2.32	0.06	0.46	2.16	3.79	4.21	0.15											3.05	100.4	8	5				0.5		7	20	0.110
CJ-3	Upper crust	Gangdese, China	Altered granite porphyry	69.1	0.38	15.95	2.43	0.1	0.45	1.29	4.41	3.67	0.15											2.24	100.28	10	4				0		2	733	0.030
BR-2	Upper crust	Gangdese, China	Biotite monzonite porphyry	70.1	0.37	15.65	1.77	<0.01	0.7	0.7	5.37	3.46	0.14											1.09	99.51	4	1				0.8		5	461	0.030
GJ-3	Upper crust	Gangdese, China	Tonalite porphyry	65	0.43	16.05	3.44	0.03	1.37	1.78	5.51	3.49	0.17											0.72	98.17	17	5				0		16	217	0.040
GJ-1	Upper crust	Gangdese, China	Granite porphyry	69.2	0.32	15.05	0.89	0.02	0.64	1.27	5.36	3.89	0.11											2.21	99.11	4	3				0		2	263	0.050
GJ-4	Upper crust	Gangdese, China	Diorite	60	0.92	13.55	4.75	0.07	5.61	3.27	3.09	6.23	0.58											0.87	99.27	183	17				0.7	1	1	238	0.060
PB-2	Upper crust	Gangdese, China	Mafic dyke	51.6	1.18	17.4	9.33	0.14	3.71	7.67	4.42	1.68	0.52											0.7	98.52	1	18				0		1	72	0.020
CJ-4	Upper crust	Gangdese, China	A-vein bearing granite porphyry	68.7	0.34	14.5	2.09	0.07	0.62	1.67	0.13	5.96	0.12											3.8	98.08	5	5				0		47	617	0.670
TG-3	Upper crust	Gangdese, China	K-feldspar porphyritic tonalite	65.9	0.45	15.1	2.66	0.06	1.06	2.67	3.88	3.63	0.17											2.39	98.15	10	5				0		4	100	0.060
CJ-1	Upper crust	Gangdese, China	Monzogranite porphyry	67.2	0.41	15.25	2.65	0.05	0.99	1.43	4.3	3.45	0.17											2.26	98.41	7	5				0		3	4	0.170
SNM-1	Upper crust	Gangdese, China	Metamorphic gabbro with skarn veins	47.6	0.78	18.05	10.8	0.17	6.72	11.4	2.28	0.69	0.16											0.39	99.14	27	36				3.6	6	9	95	0.010
TG-2	Upper crust	Gangdese, China	K-feldspar-plagioclase porphyritic tonalite	69.5	0.52	16.5	2.86	0.01	1.41	1.59	4.63	3.51	0.19											1.53	102.25	9	6				0		25	634	0.010
G318-6	Upper crust	Gangdese, China	Lamprophyre dyke	47.1	1.14	10.4	7.58	0.13	9.87	7.58	0.61	6.96	1.37											3.27	96.82	267	35				1.7	2	3	156	0.020
CC-16-01.1	Upper crust	Cripple Creek, CO	Phonolite + quartz-fluorite-telluride veins	54.72	0.66	17.12	4.06	0.24	1.28	2.128	1.93	11.39	0.25	1.566					0.185					1.77	97.30	3.68	7.17					60.7	270.8	8.61	1.325
CC-16-02.2	Upper crust	Cripple Creek, CO	Phonolite + quartz-fluorite-telluride veins	59.42	0.75	18.63	2.56	0.17	0.11	0.202	0.55	15.06	0.05	0.127					0.218					0.46	98.30	3.74	6.88					65.7	1933.9	12.90	3.428
CC-16-03.2	Upper crust	Cripple Creek, CO	Phonolite + quartz-fluorite-telluride veins	57.57	0.71	20.20	4.88	0.05	0.69	0.039	4.87	7.63	0.30	0.030					0.176					0.61	97.77	n.d.	3.13					76.6	24.5	4.44	0.045
CC-16-4	Upper crust	Cripple Creek, CO	Quartz-fluorite-telluride vein	55.56	0.65	20.38	4.03	0.06	0.77	1.184	0.38	13.84	0.24	0.290					0.138					0.51	98.03	1.88	3.53				5.05	126.2	651.4	5.06	1.601
VV-16-03.3	Upper crust	Cripple Creek, CO	Tephriphonolite with telluride veins	55.79	0.51	18.16	3.26	0.29	0.66	1.950	0.51	14.26	0.14	1.231					0.159					0.34	97.27		2.64					125.6	45.7	3.10	0.895
VV-16-07.1	Upper crust	Cripple Creek, CO	Tephriphonolite with telluride veins	56.91	0.65	16.73	3.47	0.26	0.68	1.271	0.52	14.68	0.25	1.127					0.325					0.42	97.30	1.51	4.18					31.3	166.5	2.65	0.541
